# Does new onset and pre-existing atrial fibrillation predict mortality in COVID-19 patients?

**DOI:** 10.1186/s43044-022-00291-9

**Published:** 2022-07-07

**Authors:** Selim Aydemir, Emrah Aksakal, Faruk Aydınyılmaz, Oktay Gülcü, İbrahim Saraç, Sidar Şiyar Aydın, Remziye Doğan, Merve Lazoğlu, Kamuran Kalkan

**Affiliations:** 1 Department of Cardiology, Mareşal Çakmak State Hospital, Erzurum, Turkey; 2 Department of Cardiology, Erzurum Education and Research Hospital, University of Health Sciences, Erzurum, Turkey; 3 Department of Cardiology, Düzce Atatürk State Hospital, Düzce, Turkey; 4 Department of Midwifery, Atatürk University Faculty of Health Sciences, Erzurum, Turkey; 5 Department of Cardiology, Dışkapı Yıldırım Beyazıd Education and Research Hospital, University of Health Sciences, Ankara, Turkey

**Keywords:** COVID-19, Atrial fibrillation, Mortality

## Abstract

**Background:**

Coronavirus Disease-2019 (COVID-19), caused by Severe Acute Respiratory Syndrome-Coronavirus-2, still remains prevalent and severe. We aimed to evaluate the effects of pre-existing atrial fibrillation and new-onset atrial fibrillation (NOAF) on the clinical severity and mortality of COVID-19.

**Results:**

Between April and December 2020, 5577 patients with positive PCR and/or COVID-19 compatible findings in computed tomography hospitalized were enrolled retrospectively. Total and in-hospital mortality, need for intensive care unit (ICU), need for mechanical ventilation, and recurrent hospitalization results of 286 patients with pre-existing AF before hospitalization and 82 patients with NOAF during hospitalization were evaluated. Preexisting AF was associated with a 2-fold increase in total and in-hospital mortality [OR (2.16 (1.62–2.89), 2.02 (1.48–2.76), *P* < 0.001, respectively]. NOAF was associated with a 14-fold increase in total mortality and a 12-fold increase in in-hospital mortality [OR(14.72 (9.22–23.5), 12.56 (8.02–19.68), *P* < 0.001], respectively]. However, pre-existing AF and NOAF resulted in increased ICU admission, mechanical ventilation, and recurrent hospitalization. In the Cox regression analysis, NOAF was observed as an independent risk factor for mortality.

**Conclusions:**

Pre-existing AF and in-hospital NOAF were associated with increased mortality and severity in hospitalized COVID-19 patients. In addition, NOAF was observed as an independent prognostic indicator in terms of total mortality.

## Background

Coronavirus Disease-2019 (COVID-19) is a viral disease caused by Severe Acute Respiratory Syndrome-Coronavirus-2 (SARS-COV-2) and caused a pandemic [1]. SARS-COV-2 has led to the fact that about 215 million people have been infected worldwide and has caused the death of 4.4 million people [2]. The clinical spectrum of COVID-19 ranges from asymptomatic infection to acute respiratory distress syndrome (ARDS), multiorgan failure, and death [3]. COVID-19 can cause various clinical entities such as hypercoagulation, acute kidney injury, or cytokine storm [4]. Risk factors such as male gender, advanced age, and comorbidity are associated with COVID-19 severity [5]. Death occurs more frequently in older age or patients with comorbidities such as cardiovascular disease (CVD), arterial hypertension (HT), diabetes mellitus (DM), chronic obstructive lung disease (COPD), chronic kidney disease (CRF), obesity, or cancer. In addition, the rate of critical illness is higher among hospitalized patients, and approximately 30% of patients need intensive care [6]. Although COVID-19 mainly affects the lungs, Cardiovascular (CV) complications such as arrhythmia, acute myocardial injury, left ventricular dysfunction, acute pericarditis, venous and arterial thromboembolic events, and acute coronary syndrome may also develop. These conditions are associated with poor outcomes [7]. Atrial fibrillation (AF) has been frequently observed in COVID-19 patients [8]. The effect of this arrhythmia, which is so common and has significant clinical consequences, on the COVID-19 process has also aroused curiosity. We aimed to evaluate the impact of new-onset AF (NOAF) and pre-existing AF on COVID-19 clinical severity and mortality.

## Methods

April-December 2020, the data of 5856 hospitalized patients diagnosed with COVID-19 were retrospectively analyzed, and 5577 patients, whose records are fully accessible, enrolled in the study. This study was conducted with the approval of the local ethics committee (Approval Number: 2021-2/28) and in accordance with the Helsinki Declaration. Because of the retrospective design, informed consent was not obtained.

Patient complaints, previous medical history, comorbidities, history of drug use, and laboratory parameters were determined from electronic medical records. The results containing laboratory parameters of the blood sample taken within the first 24 h after admission were examined. Electrocardiographs (ECG) were taken at the time of admission to the hospital and when the patients had symptoms and were recorded by accessing the patient files. Drug treatments in the last year and mortality records were determined and collected from the National Medical Record System using the Social Security Institution website.

The patients were diagnosed with COVID-19 based on their symptoms, examination findings, imaging findings, blood parameters, and PCR test results [9]. Patients with negative PCR tests and no findings consistent with COVID-19 on CT were excluded from the study.

Pre-existing AF was defined as patients who already used oral anticoagulants and had documented AF before hospitalization, regardless of AF type (paroxysmal, persistent, permanent, or permanent). NOAF was defined as occurs during hospitalization due to COVID-19 and lasting more than 30 s on the monitor, regardless of the duration of the arrhythmia or the presence and severity of symptoms [10].

The patients' data were recorded after an average of 6 months of follow-up. The endpoints of the study were determined as total mortality, in-hospital mortality, need for ICU admission and mechanical ventilation, and recurrent hospitalization.

### Statistical analysis

Statistical analyses were performed using SPSS 23.0 software (SPSS Inc., Chicago, IL, USA). All continuous variables were expressed as mean ± standard deviation (SD) or median (interquartile range [IQR]). Baseline features were evaluated by appropriate statistical tests; *t*-test for continuous variables with normal distribution, Mann–Whitney *U* test for continuous variables with non-normal distribution, chi-square for categorical variables, and ANOVA for parametric variables with three independent groups. The Kruskal–Wallis test was used to analyze the three groups that did not have a normal distribution. Regression analysis was performed for statistically significant parameters obtained from the univariate analysis, and independent predictors of 6 month mortality were examined. The effect of AF types on total mortality in the entire cohort in patients with Pre-existing AF, patients with NOAF, and patients with non-AF was investigated by using Kaplan–Meier analysis. A *P*-value ≤ 0.05 was considered significant.

## Results

The mean age of 5577 patients included in our study was 61.4 ± 16.4, and 49.8% were male. 286 patients had pre-existing AF (5.1%), and 82 patients had NOAF (1.5%). The incidence of pre-existing and NOAF increased with increasing age and was more common in males. PCR test was positive in 3809 (68.3%) patients, and radiological signs of infection were observed in 4753 (85.2%) patients. Hypertension (HT), coronary artery disease (CAD), heart failure (HF), COPD, and cerebrovascular events (CVE) were observed more frequently in patients with pre-existing AF and NOAF. In addition, Ferritin, D-dimer, c-reactive protein (CRP) troponin, N-terminal pro-brain natriuretic peptide (NTproBNP) were higher, especially in NOAF patients. Baseline demographic data and laboratory parameters of the study population are shown in Table 1.

**Table 1:** Baseline characteristics of patients

Characteristics	All patients (*n* = 5577)	AF none (group 1) (*n* = 5209)	Pre-existing AF (group 2) (*n* = 286)	New-onset AF (group 3) (*n* = 82)	*P* value
Age (year)	61.4 ± 16.4	63 (50–73)	73 (66–80)	79 (72.7–83.2)	< 0.001^a,b,c^
Gender (Male, %)	2777 (49.8)	2581 (49.5)	144 (50.3)	52 (63.4)	0.044^b^
HT (number, %)	2760 (49.5)	2458 (47.2)	247 (86.4)	55 (67.1)	< 0.001^a,b,c^
DM (number, %)	1505 (27)	1404 (26.9)	88 (30.8)	13 (15.9)	0.027^c^
CAD (number, %)	1188 (21.3)	990 (19)	166 (58)	32 (39)	< 0.001^a,b,c^
HF (number, %)	294 (5.3)	183 (3.5)	94 (32.6)	17 (20.7)	< 0.001^a,b,c^
COPD (number, %)	765 (13.7)	649 (12.5)	101 (35.3)	15 (18.3)	< 0.001^a,c^
CVE (number, %)	125 (2.2)	105 (2)	17 (5.9)	3 (3.7)	< 0.001^a^
HL (number, %)	686 (12.3)	638 (12.2)	35 (12.2)	13 (15.8)	0.590
CRF (number, %)	146 (2.6)	126 (2.4)	15 (5.2)	5 (6.1)	0.002^a^
Asthma (number, %)	228 (4.1)	219 (4.2)	8 (2.8)	1 (1.2)	0.211
*Medications*					
ASA (number, %)	1210 (21.8)	1122 (21.5)	65 (22.7)	23 (28)	0.331
P2Y12 inhibitors (number, %)	357 (6.4)	313 (6)	36 (12.6)	8 (9.8)	< 0.001^a^
Anticoagulants (number, %)	1192 (24.4)	1040 (20)	131 (45.7)	21 (25.6)	< 0.001^a,b,c^
Statins (number, %)	607 (1.9)	531 (10.2)	63 (22)	13 (15.9)	< 0.001^a^
ACEI/ARB (number, %)	1908 (34.2)	1713 (32.9)	164 (57.3)	31 (37.8)	< 0.001^a,c^
BB (number, %)	1159 (20.8)	960 (18.4)	172 (60.1)	27 (32.9)	< 0.001^a,b,c^
CCB (number, %)	972 (17.4)	838 (16.1)	111 (38.8)	23 (28)	< 0.001^a,b^
*Laboratory*					
Hb (g/dL)	13.3 (12.1–14.4)	13.5 (12.1–14.4)	12.9 (11.3–14.4)	12.5 (10.6–14.0)	< 0.001^a,b^
Wbc (10^3^/µL)	7.21 (5.53–9.55)	7.12 (5.51–9.42)	8.17 (5.99–10.76)	10 (7.5–13.1)	< 0.001^a,b,c^
Platelet count (10^3^/µL)	231.7 (184–289.2)	233 (186–291)	205 (159–262)	190 (132–261)	< 0.001^a,b^
AST (U/L)	33 (24.5–47.5)	32.7 (24.5–47)	33.2 (25.6–46.4)	52.7 (31.9–103.6)	< 0.001^a,b^
ALT (U/L)	31.3 (21–49.5)	31.5 (21.3–50)	27.5 (18.3–39.1)	39 (23.2–73.4)	< 0.001^b,c^
Ferritin, (ng/mL)	257.4 (111.4–547.7)	256.4 (110.9–539.1)	243.8 (95.7–506.2)	645.3 (229.4–1019.2)	< 0.001^b,c^
CRP (mg/L)	36.8 (12.8–75.7)	36.2 (12.5–74.7)	35.7 (13.4–77.2)	85.8 (37–142.7)	< 0.001^b,c^
D-dimer (μg/mL)	384 (104–1318)	363 (100–1238)	666 (189–2100)	3131 (1182–6574)	< 0.001^a,b,c^
Procalcitonin (ng/mL)	0.11 (0.02–0.57)	0.10 (0.02–0.54)	0.19 (0.03–0.94)	0.94 (0.27–5.50)	< 0.001^b,c^
Troponin I (ng/mL)	0.011 (0.003–0.1)	0.01 (0.002–0.085)	0.04 (0.01–0.14)	0.41 (0.07–1.24)	< 0.001^b,c^
NTproBNP (pg/mL)	444 (83.8–4916.5)	353.5 (71.2–3497.2)	6781 (2136–20,947)	8595 (954–15,745)	< 0.001^a,b^
Creatinine (mg/dL)	0.88 (0.73–1.12)	0.87 (0.73–1.10)	1.12 (0.86–1.51)	1.23 (0.88–1.76)	< 0.001^a,b^
Na (mmol/L)	137 (134.6–139.1)	137 (134–139)	137 (134–140)	139 (135–143)	< 0.001^b,c^
K (mmol/L)	4.18 (3.89–4.49)	4.18 (3.89–4.48)	4.21 (3.91–4.57)	4.45 (4.11–4.78)	< 0.001^a,b^
Ca (mg/dL)	8.6 (8.3–8.9)	8.7 (8.32–9.05)	8.6 (8.23–8.9)	8.12 (7.79–8.5)	< 0.001^a,b,c^
Mg (mg/dL)	1.86 (1.72–1.98)	1.87 (1.73–2.02)	1.83 (1.68–1.96)	1.99 (1.74–2.21)	0.382
Glucose (mg/dL)	131 (136–179.3)	130 (105–177)	143 (112–185)	163 (133–199)	< 0.001^b^
COVID-19 PCR test (positive, %)	3809 (68.3)	3602 (69.1)	156 (54.5)	51 (62.2)	< 0.001^a^
Lung involvement in tomography (positive, %)	4753 (85.2)	4452 (85.5)	234 (81.8)	67 (81.7)	0.158
*Disease progression*					
Total mortality (number, %)	754 (13.5)	633 (12.2)	66 (23.1)	55 (67.1)	< 0.001^a,b,c^
In-hospital mortality (number, %)	627 (11.2)	526 (10.1)	53 (18.5)	48 (58.5)	< 0.001^a,b,c^
Need for intensive care (number, %)	897 (16.1)	766 (14.7)	79 (27.6)	52 (63.4)	< 0.001^a,b,c^
Need for intubation (number, %)	493 (8.8)	410 (7.9)	43 (15)	40 (48.8)	< 0.001^a,b,c^
Recurrent hospitalization (number, %)	579 (10.4)	518 (9.9)	45 (15.7)	16 (19.5)	< 0.001^a,c^
Total hospital stay (day)	8 (5–13)	8 (5–13)	9 (5–14)	17 (9–26)	< 0.001^b,c^

Abbreviations: *AF* atrial fibrillation *HT* hypertension, *DM* diabetes mellitus, *CAD* coronary artery disease, *HF* heart failure *COPD* chronic obstructive pulmonary disease, *CVE* cerebrovascular event, *HL* hyperlipidemia, *CRF* chronic renal failure, *ASA* acetylsalicylic acid *ACEI* angiotensin-converting enzyme inhibitor, *ARB* angiotensin receptor blocker, *BB* beta-blocker, *CCB* calcium channel blockers, *Hb* hemoglobin, *Wbc* white blood cell, *AST* aspartate aminotransferase, *ALT* alanine aminotransferase, *CRP* C-reactive protein, *NTproBNP* N-terminal pro-brain natriuretic peptide, *Na* sodium, *K* potassium, *Ca* calcium, *Mg* magnesium, *PCR* polymerase chain reaction

^a^Statistical difference between Group 1 vs. Group 2

^b^Statistical difference between Group 1 vs. Group 3

^c^Statistical difference between Group 2 vs. Group 3

Considering the study's endpoints, the total mortality rate in the patients was 13.5%, and the in-hospital mortality was 11.2%. The rate of patients needing intensive care was 16.1%, the rate of intubation was 8.8%, and the rate of recurrent hospitalization was 10.4%. Total mortality rate in patients without AF, pre-existing AF and NOAF was 12.2% (633), 23.1% (66), 67.1% (55), in-hospital mortality rate 10.1% (526), 18.5% (53), 58.5% (48), need for intensive care unit 14.7% (766), 27.6% (79), 63.4% (52) mechanical ventilation need 7.9% (410), 15 (43), 48.8% (40), recurrent hospitalization rate was 9.9% (518), 15.7% (45), 19.5% (16), mean hospital stay was 8 days, 9 days, 17 days (respectively).

Pre-existing AF was associated with a 2-fold increase in total and in-hospital mortality [OR (2.16 (1.62–2.89), 2.02 (1.48–2.76), *P* < 0.001], respectively). Likewise, it was associated with an increased need for ventilation and recurrent hospitalizations [OR (2.21, 2.07, 1.69, *P* < 0.001, respectively). It was found that NOAF increased total mortality 14 times and in-hospital mortality 12 times [OR(14.72 (9.22–23.5), 12.56 (8.02–19.68), *P* < 0.001]). It was also observed that the need for ventilation and the need for re-hospitalization increased significantly [OR(10.05, 11.14, 2.19, respectively, *P* < 0.001)] (Tables 2 and 3).

**Table 2:** Effects of AF types on the course of the disease

	Pre-existing AF OR (%95 CI)	*P* value	New-onset AF OR (%95 CI)	*P* value
Total mortality	2.16 (1.62–2.89)	< 0.001	14.72 (9.22–23.5)	< 0.001
In-hospital mortality	2.02 (1.48–2.76)	< 0.001	12.56 (8.02–19.68)	< 0.001
Need for intensive care	2.21 (1.68–2.90)	< 0.001	10.05 (6.37–15.86)	< 0.001
Need for intubation	2.07 (1.47–2.90)	< 0.001	11.14 (7.14–17.38)	< 0.001
Recurrent hospitalization	1.69 (1.21–2.35)	0.002	2.19 (1.26–3.81)	< 0.001

Abbreviations: *AF* atrial fibrillation

**Table 3:** Cox-regression analysis on total mortality

	Univariate OR, 95 CI	*P*	Multivariate OR, 95 CI	*P*
*Cox-regression analysis of comorbid diseases on total mortality*				
Age	1.07 (1.06–1.08)	< 0.001	1.07 (1.06–1.08)	< 0.001
Gender (female)	0.61 (0.52–0.70)	< 0.001	0.61 (0.52–0.70)	< 0.001
HT	1.93 (1.66–2.24)	< 0.001	0.99 (0.84–1.18)	0.973
DM	1.18 (1.01–1.38)	0.042	1.07 (0.91–1.26	0.369
CAD	1.63 (1.40–1.91)	< 0.001	0.94 (0.79–1.12)	0.537
HF	2.44 (1.95–3.07)	< 0.001	1.36 (1.05–1.76)	0.018
COPD	2.10 (1.77–2.48)	< 0.001	1.25 (1.05–1.49)	0.009
CVE	1.53 (1.03–2.29)	0.034	0.91 (0.61–1.37)	0.676
CRF	2.36 (1.73–3.23)	< 0.001	1.80 (1.31–2.47)	< 0.001
AF	2.52 (2.21–2.87)	< 0.001	1.56 (1.35–1.79)	< 0.001
*Cox-regression analysis of comorbid diseases and AF types on total mortality*				
Age	1.07 (1.06–1.08)	< 0.001	1.07 (1.06–1.08)	< 0.001
Gender (female)	0.61 (0.52–0.70)	< 0.001	0.61 (0.52–0.70)	< 0.001
HT	1.93 (1.66–2.24)	< 0.001	0.99 (0.84–1.18)	0.973
DM	1.18 (1.01–1.38)	0.042	1.07 (0.91–1.26	0.369
CAD	1.63 (1.40–1.91)	< 0.001	0.94 (0.79–1.12)	0.537
HF	2.44 (1.95–3.07)	< 0.001	1.36 (1.05–1.76)	0.018
COPD	2.10 (1.77–2.48)	< 0.001	1.25 (1.05–1.49)	0.009
CVE	1.53 (1.03–2.29)	0.034	0.91 (0.61–1.37)	0.676
CRF	2.36 (1.73–3.23)	< 0.001	1.80 (1.31–2.47)	< 0.001
Pre-existing AF	1.90 (1.48–2.45)	< 0.001	1.08 (0.83–1.43)	0.539
NOAH	6.90 (5.24–9.08)	< 0.001	2.98 (2.24–3.96)	< 0.001

Abbreviations: *HT* hypertension, *DM* diabetes mellitus, *CAD* coronary artery disease, *HF* heart failure, *COPD* chronic obstructive pulmonary disease, *CVE* cerebrovascular event, *CRF* chronic renal failure, *AF* atrial fibrillation, *NOAF* new-onset atrial fibrillation

**Fig. 1:**
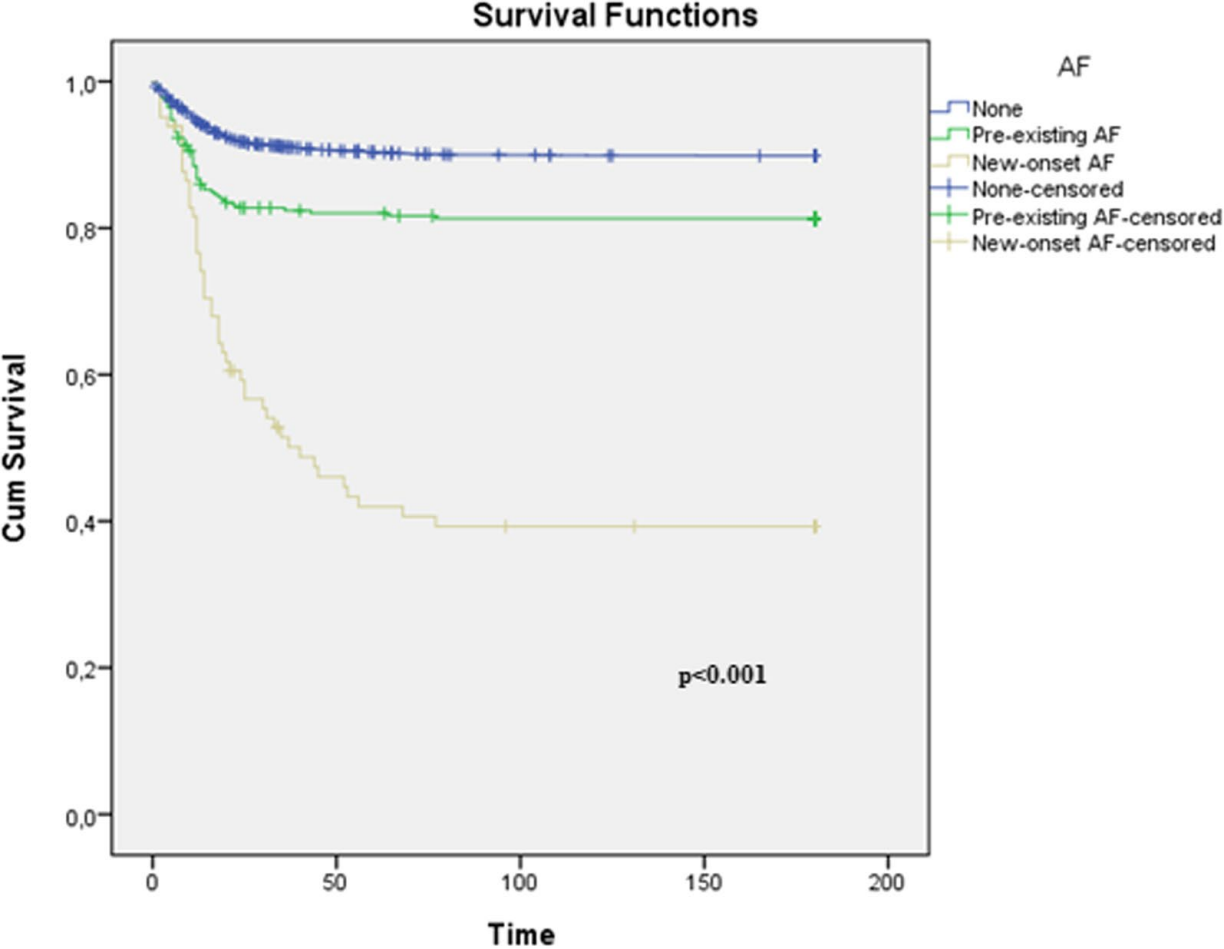
Survival curves according to AF types

The regression analysis, performed to investigate the effect of AF and other comorbid diseases on total mortality, determined that AF increased mortality 1.5 times. In the analysis of which of the AF subtypes the increase in mortality was related to, it was observed that this increase was caused by NOAF and was an independent risk factor in predicting mortality. In addition, the effects of non-AF, NOAF, and pre-existing AF on survival in Kaplan–Meier analysis are shown in Fig. 1.

## Discussion

Our study revealed that there was a significant increase in total and in-hospital mortality, need for intensive care, requiring mechanical ventilation, and recurrent hospitalizations with NOAF and pre-existing AF in hospitalized COVID-19 patients.

AF is more common in those with comorbidities such as male gender, advanced age, heart failure (HF), HT, coronary artery disease (CAD), heart valve disease, cerebrovascular events (CVE), obesity, DM, and CRF [11]. In addition, the frequency of NOAF increases in patients with ARDS and sepsis, as well as in patients with viral respiratory infections [12]. Similarly, comorbidities with pre-existing AF and NOAF were more common in our study.

The prevalence of arrhythmias in COVID-19 patients is variable [8]. However, arrhythmias associated with COVID-19 are mostly atrial. In a study, atrial arrhythmia was observed in 15.8% of the patients, and 9.6% were newly diagnosed [13]. In another study, arrhythmia was observed in 18.5% of patients requiring invasive mechanical ventilation, and atrial arrhythmia was observed in 95.8% of them [14]. Various data are available on the prevalence of AF in COVID-19, and it is considered the most common arrhythmia to occur. The proportion of patients hospitalized for COVID-19 with a history of AF varies between 5 and 25% [8, 15]. However, there are limited reliable data on NOAF in COVID-19 patients, and its prevalence ranges from 3.5 to 5% [16]. In addition, it has been shown that the frequency of AF increases in ICU patients with COVID 19 [17]. In our study, 5.1% of COVID-19 patients had pre-existing AF, and NOAF developed in 1.4%.

Although the exact mechanisms of AF remain unclear, ion channel dysfunction, Ca (2 +) processing abnormalities (handling abnormalities), structural remodeling, and autonomic dysfunction play a role in its pathophysiology. The pathophysiology of cardiac manifestations and arrhythmias in COVID-19 is uncertain, but the mechanisms explaining the pathogenesis are direct viral myocardial damage, hypotension, and angiotensin-converting enzyme 2 (ACE2) receptor downregulation, increased inflammatory state, increased endogenous catecholamine and adrenergic status, and electrolyte abnormalities [18]. SARS-CoV-2 uses ACE2 receptors to enter host cells, including type 2 pneumocytes, cardiomyocytes, pericytes, macrophages, and endothelial cells. ACE2 also plays an important role in angiotensin II regulation and protects against the detrimental effect of the renin–angiotensin–aldosterone system (RAAS) on the cardiovascular system [19]. RAAS has an important role in the development of AF. RAAS modulates the electrical and mechanical properties of the atrium. RAAS dysregulation causes the release of angiotensin II further contributing to AF [20]. It can be thought that SARS-CoV-2 may induce AF by its effect on ACE2 and RAAS. Increased metabolic stress resulting from systemic infections and sepsis may lead to the development of AF [12]. In addition, patients hospitalized for pneumonia have an increased risk of developing AF. Furthermore, it is believed that the increased inflammatory state and cytokine storm observed in COVID-19 patients lead to the development of AF. In another study, it was suggested that SARS-COV-2 virus pericytes could contribute to the pathogenesis of AF by binding, causing inflammation of the heart tissue, and altering atrial cellular electrophysiology [21].

Regardless of COVID-19, AF is associated with high mortality and risk of CV complications in the general population. AF is a predictor of mortality for patients presenting with myocardial infarction, HF, and ischemic stroke, and the presence of comorbidities are associated with worse outcomes in patients with AF [22]. In addition, AF increases mortality, especially in patients hospitalized for sepsis [23]. However, the development of AF in severe COVID-19 patients is thought to increase the risk of adverse events in patients. Various studies have been conducted on the relationship between COVID-19 and AF. Some studies have shown that AF is associated with an increased risk of death in COVID-19 patients [13]. The presence of AF in COVID-19 is associated with the need for mechanical ventilation and increased mortality, especially in those with more serious diseases [24]. Similarly, in our study, it was observed that both pre-existing AF and NOAF significantly increased total and in-hospital mortality, intensive care hospitalization, and the need for mechanical ventilation. Unlike our study, in some studies, it was observed that AF did not affect in-hospital mortality in COVID-19 [16].

Advanced age and male gender have been shown to be predictors of mortality in COVID-19. In a meta-analysis, it was shown that advanced age and male gender are associated with HT and severe disease and increase all-cause mortality in patients with AF [25]. Similarly, advanced age and male gender were observed more frequently in pre-existing AF and NOAF patients in our study, and comorbidities such as HT, CAD, HF, COPD, and CVE were observed at a higher rate. Our study is consistent with research suggesting that AF is a risk factor independent of age and comorbidities [26].

Thromboembolic complications resulting in death have increased in COVID-19 patients. The high level of D-dimer, which indicates increased hypercoagulation, is associated with an increase in infection, sepsis, and mortality in COVID-19 patients. In addition, increased inflammation during COVID-19 has been associated with more severe disease and an increased risk of death [27]. Elevated levels of CRP and interleukin-6 (IL-6) were observed in AF patients accompanying COVID-19, indicating increased inflammation [28]. In our study, ferritin, troponin, and NTproBNP were found to be high as well as high CRP and D-dimer in pre-existing AF and NOAF patients, which shows the effect of increased inflammation on mortality.

In addition to inflammatory and comorbid conditions, AF is associated with hemodynamic instability and thromboembolism, and the presence of AF can directly worsen the prognosis [29]. In another study, it has been shown that patients who develop NOAF in COVID-19 have a high risk of embolic events [30]. The thromboembolic event data in our study are limited, but embolic complications may be one of the reasons for the increased mortality in NOAF and pre-existing AF.

## Conclusions

We observed an increase in disease severity and mortality in patients with NOAF and pre-existing AF hospitalized due to COVID-19. We determined that the prognosis was worse and was an independent predictor of mortality, especially in patients who developed NOAF.

### Limitations

Our study has some limitations. Retrospective design is the main limitation of this study, there may be hidden confusion, and the reliability of the results is questionable. Moreover, our study is observational and therefore cannot show the cause-effect relationship between the clinical prognosis of COVID-19 patients, and it needs to be confirmed by randomized controlled trials. Second, there is a lack of long-term ECG monitoring, and silent AF is likely to go unnoticed. Again, echocardiography data could not be reached. Third, being a single-center study, it may not apply to other populations because it is represented by a population with a specific geographic region and socio-economic conditions. Fourth, hospitalized patients due to COVID-19 were included in the study, so the generalizability of our findings to asymptomatic or symptomatic COVID-19 patients who were not hospitalized may be limited. However, we believe that our findings contribute to chronic and AF prevalence data in COVID-19 patients, identifying risk factors causing the development of NOAF, and its impact on the mortality and severity of COVID-19.

## Data Availability

The datasets used and analyzed during the current study are available from the corresponding author on reasonable request.

## Abbreviations

COVID-19: Coronavirus Disease-2019 (COVID-19),
SARS-CoV-2: Severe Acute Respiratory Syndrome-Coronavirus-2
AF: Atrial fibrillation
NOAF: New-onset atrial fibrillation
CT: Computed tomography
ICU: Intensive care unit
ARDS: Acute respiratory distress syndrome
CVD: Cardiovascular disease
HT: Arterial hypertension
DM: Diabetes mellitus
COPD: Chronic obstructive lung disease
CRF: Chronic kidney disease
ECG: Electrocardiography
CRP: C-reactive protein
NTproBNP: N-terminal probrain natriuretic peptide
CAD: Coronary artery disease
CVE: Cerebrovascular accident
ACE2: Angiotensin-converting enzyme 2
RAAS: Renin-angiotensin-aldosterone system
HF: Heart failure
IL: Interleukin
